# Drug survival superiority of tumor necrosis factor inhibitors and interleukin-17 inhibitors over Janus kinase inhibitors and interleukin-12/23 inhibitors in German psoriatic arthritis outpatients: retrospective analysis of the RHADAR database

**DOI:** 10.3389/fimmu.2024.1395968

**Published:** 2024-05-23

**Authors:** Patrick-Pascal Strunz, Matthias Englbrecht, Linus Maximilian Risser, Torsten Witte, Matthias Froehlich, Marc Schmalzing, Michael Gernert, Astrid Schmieder, Peter Bartz-Bazzanella, Cay von der Decken, Kirsten Karberg, Georg Gauler, Patrick Wurth, Susanna Späthling-Mestekemper, Christoph Kuhn, Wolfgang Vorbrüggen, Johannes Heck, Martin Welcker, Stefan Kleinert

**Affiliations:** ^1^ Department of Medicine II, Rheumatology/Clinical Immunology, University Hospital of Wuerzburg, Würzburg, Germany; ^2^ Independent Researcher, Greven, Germany; ^3^ Department of Rheumatology and Immunology, Medical School Hannover, Hannover, Germany; ^4^ Clinic for Dermatology, Venereology and Allergology, University Hospital Wuerzburg, Wuerzburg, Germany; ^5^ Klinik für Internistische Rheumatologie, Rhein-Maas-Klinikum, Würselen, Germany; ^6^ Medizinisches Versorgungszentrum, Stolberg, Germany; ^7^ Verein zur Förderung der Rheumatologie e.V., Würselen, Germany; ^8^ Rheumatologisches Versorgungszentrum Steglitz, Berlin, Germany; ^9^ Private Practitioner, Osnabrück, Germany; ^10^ Rheumapraxis München, Munich, Germany; ^11^ Praxis für Rheumatologie, Karlsruhe, Germany; ^12^ Institute for Clinical Pharmacology, Hannover Medical School, Hannover, Germany; ^13^ Medizinisches Versorgungszentrum für Rheumatologie Dr. M. Welcker GmbH, Planegg, Germany; ^14^ Praxisgemeinschaft Rheumatologie-Nephrologie, Erlangen, Germany

**Keywords:** treatment persistence, tofacitinib, upadacitinib, ustekinumab, real-world, biologics, Kaplan-Meier analysis, psoriasis vulgaris

## Abstract

**Objective:**

Treatment options with disease-modifying antirheumatic drugs (DMARDs) for psoriatic arthritis (PsA) have evolved over recent years. In addition to Janus kinase inhibitors (JAKi), four classes of biologic DMARDs (bDMARDs; interleukin [IL]-23 inhibitors [IL-23i], IL-12/23 inhibitors [IL-12/23i], tumor necrosis factor inhibitors [TNFi], and IL-17 inhibitors [IL-17i]) are currently approved for moderate to severe PsA treatment. There is minimal evidence of the persistence of these drugs among PsA outpatients in a real-world scenario during the period following the approval of JAKi. Therefore, we aimed to analyze the drug survival rates of biologic and JAKi therapies among German PsA outpatients during routine clinical care.

**Methods:**

We retrospectively analyzed PsA patients with a new prescription for a biologic or JAKi in the RHADAR database between January 2015 and October 2023. Kaplan-Meier Curves and Cox regression modelling were used to compare drug survival rates.

**Results:**

1352 new prescriptions with bDMARDs (IL-12/23i [n=50], IL-23i [n=31], TNFi [n=774], IL-17i [n=360]) or JAKi (n=137) were identified. The 5-year drug survival rate was 67.8% for IL-17i, 62.3% for TNFi, 53.3% for JAKi, and 46.0% for IL-12/23i. Discontinuation probabilities for JAKi and IL-12/23i were significantly higher compared with TNFi (JAKi hazard ratio [HR] 1.66, [95% CI 1.23–2.24], p=0.001; IL-12/23i HR 1.54, [95% CI 1.02–2.33], p=0.042) and IL-17i (JAKi HR 1.77, [95% CI 1.27–2.47], p=0.001; IL-12/23i HR 1.64, [95% CI 1.06–2.55], p=0.027). JAKi-treated patients had more severe disease and more osteoarthritis (OA) compared to TNFi and more OA compared to IL-17i.

**Conclusion:**

German PsA outpatients might persist longer with TNFi and IL-17i compared with IL-12/23i or JAKi. For TNFi, differences in subgroup characteristics and comorbidities (OA) may have affected drug survival rates. For IL-17i, the longer drug survival might not only be related to less OA compared to JAKi and, therefore, might be affected by other factors.

## Introduction

Over the past decade, there has been a remarkable evolution in disease-modifying antirheumatic drug (DMARD) treatment options for patients with psoriatic arthritis (PsA): As of February 2024, agents with five major modes of action (MoA) within the categories of biologic DMARDs (bDMARDs) and targeted synthetic DMARDs (tsDMARDs) have gained approval for the treatment of moderate to severe forms of PsA (not including apremilast and abatacept) (1, 2). These MoAs encompass tumor necrosis factor inhibitors (TNFi), interleukin (IL)-17 inhibitors (IL-17i), IL-12/23 inhibitors (IL-12/23i), IL-23 inhibitors (IL-23i), and Janus kinase inhibitors (JAKi) (1, 2).

Beyond evaluating treatment response through disease activity measures and response criteria, such as the American College of Rheumatology 50% improvement criteria (ACR50), the consideration of treatment survival is also essential when assessing and comparing treatment effectiveness (3–5). “Drug survival” is the term introduced to depict the continued use of a specific medication over time and serves as a surrogate indicator for both efficacy and safety in routine clinical practice (4, 5).

While there has been extensive research on the drug survival of TNFi, IL-17i, and IL-12/23i in PsA and psoriasis vulgaris (PsO) over the last decade, sufficient data for JAKi and IL-23i, the most recently approved drug classes, are not yet available (4–8). Furthermore, results from assessments conducted in PsO and other forms of inflammatory arthritis may vary from those in PsA (5). Accordingly, data from other conditions may not accurately apply to PsA.

Conducting drug survival studies in a real-world outpatient care setting, as opposed to a specialized trial center, is challenging due to the difficulty in accessing adequate data (9). The advent of digitalization in medicine and the development of databases offer promising tools for aggregating and analyzing patient data, particularly in diseases with a rather low prevalence such as PsA (9).

The RHADAR network was established to address this need for real-world data in patients with rheumatic diseases. This network currently comprises seven rheumatic practices and one outpatient department of a rheumatologic hospital in Germany. These entities contribute pseudonymized data for aggregation into the RHADAR joint database (9).

The objective of this study was to utilize data from the RHADAR database to evaluate the drug survival of JAKi compared with bDMARDs in German outpatients undergoing treatment for PsA within a real-world context.

## Methods

### Study design

A retrospective analysis of the RHADAR database for PsA patients initiating treatment with an IL-12/23i, IL-23i, TNFi, IL-17i, or JAKi medication between 15-JAN-2015 and 17-OCT-2023 was conducted. All available TNFi (adalimumab, certolizumab, etanercept, golimumab, and infliximab), IL-17i (ixekizumab, secukinumab), IL-12/23i (ustekinumab), IL-23i (risankizumab, guselkumab), and JAKi (tofacitinib and upadacitinib) were considered and aggregated according to their MoA. Bimekizumab was not considered in the analysis due to the short time between its approval and the retrospective analysis period.

### Participating centers

During the study period, there were 22 board certified rheumatologists in seven German practices and one German outpatient department of a hospital specialized in rheumatology affiliated with the RHADAR database (9). These entities permanently contributed pseudonymized data for aggregation into the RHADAR joint database (9).

### Participants

All patients had a confirmed diagnosis of PsA by these board-certified rheumatologists. Demographic characteristics and disease activity measures, including the Disease Activity in Psoriatic Arthritis (DAPSA) score (higher scores indicating higher disease activity), the patient global assessment of disease activity (PtGA, range 0 to 100 with higher scores indicating higher disease activity), joint counts, and the Funktionsfragebogen Hannover (FFbH, range 0 to 100 with higher scores indicating higher functionality capacity) functional assessment, were based on data collected during routine clinical care as determined by the treating rheumatologist. All patients in Germany have either private or public health insurance, both paying equally for biologic or targeted synthetic DMARDs.

### Ethical approval

All participants provided written informed consent to be included in the RHADAR database and allow their pseudonymized data to be analyzed. This study was submitted to the ethics committee of the Medical Faculty of the University of Wuerzburg (Application 20240122 03). The ethics committee considered the study a retrospective data analysis of pseudonymzied collected clinical routine data that did not require ethical approval by German law as long as publication of data is conducted in anonymous form.

### Statistical methods

A formal study size calculation was not performed due to the retrospective observational nature of the study. The sample size of the analysis presented was determined by the amount of available data on PsA patients in the RHADAR database during the analysis period.

Kaplan-Meier (KM) analysis was conducted to compare the drug survival of each bDMARD class and JAKi over 60 months. In this context, events were defined as cases discontinuing the respective bDMARD or JAKi treatment. Disease-related and demographic parameters, concomitant medication, and comorbidities further characterized overall and drug-class-specific study populations. Descriptive measures of sample characterization include the absolute and relative numbers of available values (n, %), arithmetic mean, standard deviation (SD), 95% confidence intervals (CI) of the mean, median, and 25^th^ and 75^th^ distribution quantiles defining the interquartile range. Results on parametric data are presented as mean (standard deviation, SD) in the text if not stated otherwise.

Cox regression was used to compare the potential impact of MoA on the risk of drug discontinuation over time, with the MoA showing the best drug persistence used as the reference. The regression models were also adjusted for age, sex, and disease duration. Hazard ratios (HRs), including respective 95% CIs, are given for each independent variable. P values <0.05 were considered significant. All calculations were conducted using the statistics software R (version 3.5.1) and RStudio (version 1.1.453) (10, 11). For distribution analysis of dichotomous variables, 95% CIs were used, and odds ratios (OR) and Chi-square tests were calculated for nominal variables. For these analyses, Prism Version 5 was used. Missing values were not imputed to retain the original information of the data available.

## Results

### Study population

In total, 1352 newly initialized bDMARD or JAKi therapies with corresponding additional patient data were identified from the RHADAR database and included in this analysis (496 males [36.7%], 855 females [63.2%], and one patient [0.1%] with non-binary gender). Patient characteristics at treatment start are shown in **Table 1**. The mean (SD) age was 53.8 years (12.3 years). Regarding the disease activity parameters, the mean DAPSA was 17.9 (16.3), the mean PtGA was 41.9 (24.1), and the mean FFbH was 76.2 (21.3) (**Table 1**).

**Table 1:** Baseline characteristics of patients (n=1352).

Characteristic	n	%	Mean	95%CI (lower)	95%CI (upper)	SD	SEM	Median	25% Quantile	75% Quantile
Gender (male)	496	36.7	NA	NA	NA	NA	NA	NA	NA	NA
Gender (female)	855	63.2	NA	NA	NA	NA	NA	NA	NA	NA
Age	1352	100.0	53.8	53.1	54.4	12.3	0.3	55.0	47.0	62.0
Disease duration (years)	1189	87.9	10.5	10.0	11.0	9.4	0.3	7.0	4.0	15.0
DAPSA	453	33.5	17.9	16.4	19.4	16.3	0.8	16.3	8.3	24.3
TJC (DAPSA)	855	63.2	5.8	5.3	6.3	7.3	0.2	4.0	0.0	8.0
SJC (DAPSA)	855	63.2	2.5	2.2	2.7	3.7	0.1	1.0	0.0	4.0
ESR (mm/h)	702	51.9	15.7	14.6	16.8	14.5	0.5	11.0	6.0	20.0
CRP (mg/dl)	779	57.6	0.8	0.2	1.4	8.2	0.3	0.3	0.1	0.4
Disease activity - patient (0–100)	792	58.6	41.9	40.2	43.5	24.1	0.9	42.0	23.0	60.0
Pain (0–100)	641	47.4	40.1	38.1	42.1	25.9	1.0	40.0	18.0	60.0
Morning stiffness (min)	612	45.3	69.0	56.2	81.8	161.7	6.5	30.0	16.0	60.0
FFbH	861	63.7	76.2	74.8	77.7	21.3	0.7	80.6	63.9	94.4

DAPSA, disease activity in psoriatic arthritis; TJC, tender joint count; SJC, swollen joint count; ESR, erythrocyte sedimentation rate; CRP, c-reactive protein; FFbH, Funktionsfragebogen Hannover; NA, not applicable.

### Treatment persistence

Among the 1352 initiated bDMARD or JAKi treatments identified, TNFi was the most common (774 cases), followed by IL-17i (360 cases), IL-12/23i (50 cases), and IL-23i (31 cases). Furthermore, 137 initiations of JAKi were recorded.

**Figure 1:**
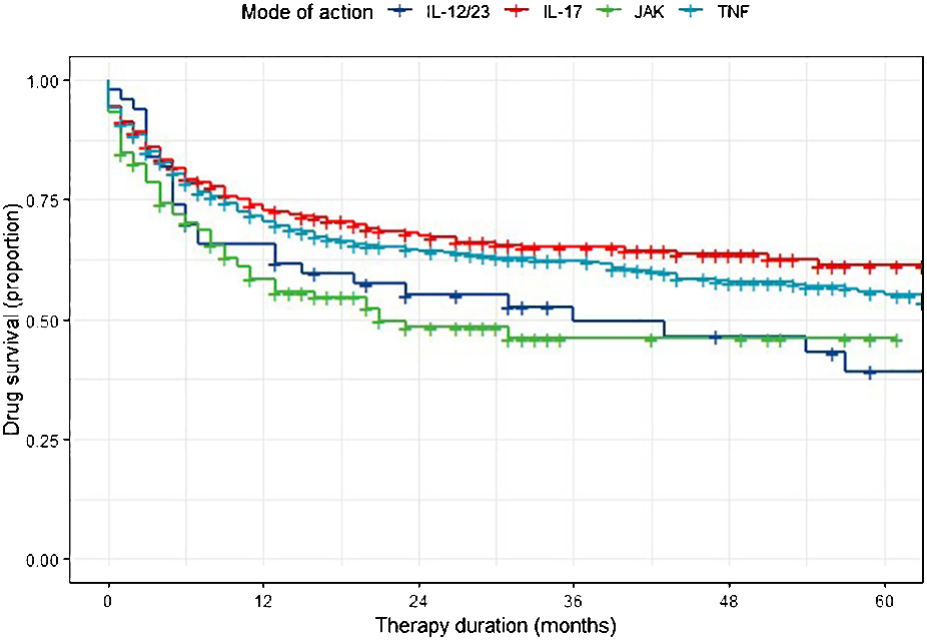
Kaplan-Meier analysis of drug persistence in PsA patients.

The KM diagram for drug survival of the different drug classes is presented in **Figure 1**. The 1-year drug survival rate was 75.0% for IL-17i, followed by 72.5% for TNFi, 66.0% for IL-IL12/23i, and 60.6% for JAKi (**Table 2**). A 5-year drug survival probability of 67.8% was found for IL-17i, 62.3% for TNFi, 53.3% for JAKi, and 46.0% for IL-12/23i. For all of the drug classes, the steepest decline in drug survival was observed during the first year of use. An analysis for IL-23i was not performed due to the small number of patients, likely due to the short time since the approval of these agents.

**Table 2:** Survival probabilities for each MoA.

	1-year	2-years	3-years	5-years
**IL-17i**	75.0% (220 + 50/360)	70.6% (145 + 109/360)	68.9% (104 + 144/360)	67.8% (35 + 209/360)
**TNFi**	72.5% (494 + 67/774)	66.4% (376 + 138/774)	64.9% (256 + 246/774)	62.3% (80 + 402/774)
**JAKi**	60.6% (64 + 19/137)	54.0% (30 + 44/137)	53.3% (7 + 66/137)	53.3% (1 + 72/137)
**IL-12/23i**	66.0% (32 + 1/50)	56.0% (23 + 5/50)	54.0% (17 + 10/50)	46.0% (9 + 14/50)

Survival probability was calculated by [n (at risk) + n (cumulative censored)]/n (at risk at month 0). The corresponding values are presented in the brackets. The first number presents patients at risk at that time point, the second number the cumulative censored events at that time point, and the last number all events at risk at month 0.

Results from Cox regression indicated that IL-12/23i (HR 1.64, [95% CI 1.06–2.55], p= 0.027) or JAKi (HR 1.77, [95% CI 1.27–2.47], p=0.001) had a higher probability of drug discontinuation compared with IL-17 as a reference. For TNFi, the probability of drug discontinuation (HR 1.07, [95% CI 0.85–1.34], p=0.587) was not different from IL-17i. Furthermore, TNFi persistence was higher than JAKi (HR 1.66, [95% CI 1.23–2.24], p=0.001) and IL-12/23i (HR 1.54, [95% CI 1.02–2.33], p=0.042), while there was no difference between persistence with JAKi and IL-12/23i (HR 0.93, [95% CI 0.57–1.49], p= 0.750). Demographic covariates in the model suggested female gender (compared to male gender as reference) (HR = 1.69, [95% CI 1.37–2.09], p<0.001) and shorter disease duration (HR = 0.99, [95% CI 0.98–1.00], p=0.033) to be independently associated with drug discontinuation.

### Patient characteristics by drug class

For each drug class, the corresponding subpopulation was descriptively analyzed to provide insights into baseline characteristics that might have influenced drug survival (**Table 3**). Because HRs were adjusted for age, sex, and disease duration, the impact of these characteristics as an explanation for different survival probabilities can be eliminated.

**Table 3:** Patient characteristics stratified by treatment MoA.

Characteristic	TNFi (n=774)		IL-17i (n=360)		IL-12/23i (n=50)		IL-23i (n=31)		JAKi (n=137)	
	n (%)	Mean (95% CI)	n (%)	Mean (95% CI)	n (%)	Mean (95% CI)	n (%)	Mean (95% CI)	n (%)	Mean (95% CI)
Male	296 (38.2)	–	137 (38.1)	–	9 (18.0)	–	13 (41.9)	–	41 (29.9)	–
Female	477 (61.6)	–	223 (61.9)	–	41 (82.0)	–	18 (58.1)	–	96 (70.1)	–
Age	774 (100)	52.8 (51.9 – 53.7)	360 (100)	54.5 (53.3 – 55.7)	50 (100)	54.1 (51.3 – 56.8)	31 (100)	54.7 (50.7 – 58.7)	137 (100)	57.1 (55.3 – 58.9)
Disease duration (yrs)	681 (88.0)	10.1 (9.4 – 10.8)	320 (88.9)	10.5 (9.5 – 11.5)	43 (86.0)	13.3 (9.8 – 16.9)	31 (100)	10.8 (8.0 – 13.5)	114 (83.2)	11.6 (9.7 – 13.5)
DAPSA	240 (31.0)	16.5 (14.1 - 18.9)	108 (30.0)	17.9 (15.5 – 20.3)	16 (32.0)	21.9 (15.4 – 28.3)	12 (38.7)	15.5 (6.2 – 24.9)	77 (56.2)	21.8 (19.0 – 24.7)
TJC (DAPSA)	476 (61.5)	5.0 (4.4 – 5.6)	223 (61.9)	6.2 (5.1 – 7.3)	28 (56.0)	9.3 (6.1 – 12.5)	19 (61.3)	5.0 (1.2 – 8.8)	109 (79.6)	7.6 (6.3 – 8.8)
SJC (DAPSA)	476 (61.5)	2.2 (1.9 - 2.5)	223 (61.9)	2.7 (2.1 – 3.3)	28 (56.0)	2.8 (1.6 – 4.0)	19 (61.3)	1.4 (0.0 – 2.8)	109 (79.6)	3.3 (2.5 – 4.0)
ESR (mm/1^st^h)	405 (52.3)	15.5 (14.1 – 17.0)	185 (51.4)	15.9 (13.8 – 18.0)	20 (40.0)	19.5 (11.7 – 27.3)	13 (41.9)	8.2 (5.0 – 11.4)	79 (57.7)	16.1 (12.9 – 19.4)
CRP (mg/dl)	435 (56.2)	0.9 (-0.1 – 2.0)	198 (55.0)	0.5 (0.4 – 0.7)	23 (46.0)	0.7 (0.4 – 1.0)	18 (58.1)	0.3 (0.2 – 0.5)	105 (76.6)	0.6 (0.4 – 0.8)
PtGA	440 (56.8)	39.2 (36.8 – 41.5)	215 (59.7)	44.1 (41.1 – 47.1)	22 (44.0)	46.5 (36.1 – 56.8)	19 (61.3)	41.2 (29.5 – 52.9)	96 (70.1)	48.3 (44.3 – 52.3)
Pain	355 (45.9)	37.3 (34.5 - 40.1)	181 (50.3)	43.4 (39.7 – 47.0)	20 (40.0)	45.0 (36.2 – 53.7)	13 (41.9)	30.1 (14.4 – 45.8)	72 (52.6)	46.1 (41.0 – 51.2)
Morning stiffness (min)	343 (44.3)	57.1 (43.4 – 70.9)	172 (47.8)	90.9 (58.9 – 123.0)	19 (38.0)	55.8 (28.8 – 82.9)	13 (41.9)	158.2 (-54.0 – 370.3)	65 (47.4)	59.5 (46.2 – 72.9)
FFbH	496 (64.1)	78.4 (76.5 – 80.3)	227 (63.1)	73.5 (70.7 – 76.3)	30 (60.0)	70.5 (63.5 – 77.5)	19 (61.3)	79.1 (68.3 – 89.9)	89 (65.0)	72.4 (68.2 – 76.6)

DAPSA, disease activity in psoriatic arthritis; TJC, tender joint count; SJC, swollen joint count; ESR, erythrocyte sedimentation rate; CRP, C-reactive protein; FFbH, Funktionsfragebogen-Hannove; PtGA, patient global assessment of disease activity.

Based on 95% confidence intervals for the mean values, TNFitreated patients showed lower DAPSA (16.5 [95% CI 14.1–18.9])and PtGA scores (39.2 [95% CI 36.8–41.5]) compared with JAKitreated patients (DAPSA 21.8 [95% CI 19.0–24.7]; PtGA 48.3 [95% CI 44.3–52.3]). Restrictively, it must be mentioned that the disease activity parameters were not equally available for all patients: While the tender joint count (TJC), the swollen joint count (SJC), C-reactive protein (CRP) and PtGA were only available in about 2/3 of the cases, the total DAPSA was only completely available for 33.5% (**Table 1**).

TNFi-treated patients had a higher mean FFbH score (78.4 [95% CI 76.5–80.3]) compared with IL-17i treated patients (73.5 [95% CI 70.7–76.3]). No further differences were found among the different drug classes.

While patients with TNFi had a mean of 1.1 previous bDMARD or tsDMARD therapies, patients with IL-17i had a mean of 1.5 previous therapies, followed by IL-12/23i treated patients with a mean of 2.1 and JAKi with a mean of 2.6 previous treatments. Patients with IL-23i had an average of 4.3 previous therapies.

### Association between comorbidities and drug survival

Comorbidities can influence treatment adherence in rheumatic diseases (12, 13), so we analyzed the MoA subpopulations for distribution of the reported comorbidities based on the database entries (**Table 4**). Osteoarthritis (OA) was significantly more prominent in the JAKi subpopulation (36.5%) than in the TNFi subpopulation (23.0%, p<0.001) and IL-17i subpopulation (24.4%, p=0.007) (**Supplementary Table S3)**. Furthermore, OA was also more often reported in the IL-12/23i subpopulation (58.0%) compared with the IL-17i (24.4%, p<0.001) and TNFi (23.0%, p<0.001) subpopulations (**Table 4**; **Supplementary Table S3**).

**Table 4:** Comorbidities by drug class.

Comorbidity^a^	TNFi (n=774)	IL-17i (n=360)	IL-12/23i (n=50)	IL-23i (n=31)	JAKi (n=137)
Osteoarthritis	178 (23.0%)	88 (24.4%)	29 (58.0%)	10 (32.3%)	50 (36.5%)
Depression	26 (3.4%)	21 (5.8%)	5 (10.0%)	2 (6.5%)	6 (4.4%)
Coronary heart disease	36 (4.7%)	18 (5.0%)	0	2 (6.5%)	0
Obesity	47 (7.4%)	28 (7.8%)	3 (6.0%)	5 (16.1%)	9 (6.6%)
Cardiovascular risk factors	334 (43.2%)	211 (58.6%)	29 (58.0%)	8 (25.8%)	61 (44.5%)
Missing values	36 (4.7%)	14 (3.9%)	1 (2.0%)	0	6 (4.4%)
No comorbidity	1 (0.1%)	2 (0.6%)	0	0	0

Data are reported as n (%). Notably, no smokers (F17) were found in the JAKi subpopulation.

a Based on ICD-10 codes: osteoporosis, M15-M19; depression, F32; coronary heart disease, I25; obesity, E66; cardiovascular risk factors, I10, E78, E11-E14; F17.

The highest obesity rates were found in the IL-23i subpopulation (16.1%), while the remaining groups had similar frequency rates (6.0 to 7.8%). Cardiovascular risk factors (CVRF) were most common in the IL-12/23i and IL-17i subgroups (58% and 58.6%, respectively), followed by JAKi (44.5%) and TNFi (43.2%). CVRFs were least common in IL-23i-treated patients (25.8%). No smokers were reported in the JAKi and IL-23i subgroups, and no coronary heart disease was recorded among IL-12/23i- and JAKi-treated patients.

### Concomitant csDMARD therapies

Concomitant medication with conventional synthetic disease modifying antirheumatic drugs (csDMARD) can affect drug survival, especially in patients being treated with TNFi (14–16). Database entries were analyzed to determine whether the bDMARDs/JAKi were administered as monotherapy or combined with a csDMARD. JAKi drugs were administered as monotherapy in 79.6% of cases, while slightly lower rates were observed for bDMARDs (77.8% for IL-17i, 77.4% for IL-23i, 74.0% for IL-12/23i, and 69.2% for TNFi). The difference between JAKi and TNFi was statistically significant (p= 0.014) (**Supplementary Table S2**). Methotrexate (MTX) was the csDMARD most commonly used in combination regimens, while leflunomide and hydroxychloroquine played a minor role as concomitant therapy, accounting for 0.3% of IL-17i and 0.4% of TNFi combination therapies.

## Discussion

In this retrospective analysis among PsA outpatients, we provide novel insights into the drug persistence rates of the major drug classes for treating moderate to severe PsA in the JAKi era. The largest number of new treatments initiated during this period were TNFi, IL-17i, and JAKi. IL-12/23i and IL-23i therapy were prescribed less frequently. The highest 5-year drug survival probability was observed for IL-17i, followed by TNFi and JAKi, while the lowest rates were seen in IL-12/23i patients. Notably, most discontinuations occurred within the first 12 months after treatment initiation with discontinuation rates subsequently decreasing. Using Cox regression models, we identified a significantly higher adjusted HR for drug discontinuation of JAKi and IL-12/23i compared to IL-17i and TNFi.

These results concerning survival on JAKi are surprising given patient preference studies showing a strong preference for oral over subcutaneous therapies (17, 18). However, when interpreting the observed JAKi and IL-12/23i drug survival probabilities compared with TNFi and IL-17i, several aspects that may have affected drug survival should be taken into account. In particular, patients treated with JAKi in our cohort had higher DAPSA and PtGA scores than TNFi-treated patients. IL-12/23i- and JAKi-treated patients had more previous treatments on average than IL-17i and TNFi-treated patients. A recent study reported similar treatment patterns: First-line treatment was still a TNFi, while IL-17i and IL-12/23i were used as second- or third-line treatments, and JAKi were predominantly found as fourth-line therapies (19). Additionally, OA, which can mimic PsA disease activity, was more prominent in the IL-12/23i and JAKi subgroups. TNFi-treated patients had a higher rate of concomitant medication with MTX compared with the JAKi subgroup. In summary, JAKi-treated patients had more severe disease, more bDMARD or tsDMARD pretreatments, and more OA compared with the TNFi subgroup. In addition, the lower persistence rate of JAKi compared with bDMARDs could be partly due to safety data from the ORAL Surveillance Study, which indicated an increased risk of serious events, including cardiovascular events and cancer, with tofacitinib (20). Based on these data, the European Medicines Agency issued recommendations that applied to all JAKi, which may have resulted in increased discontinuations of JAKi as a consequence (21). These factors, alone or in combination, might explain the lower persistence of JAKi compared with TNFi. However, the better persistence rates of IL-17i cannot be so easily explained by patient or treatment characteristics as for TNFi. While the disease activity parameters (DAPSA, SJC, TJC, PtGA, CRP) and characteristics of the patients did not differ statistically between JAKi and IL-17i, only the higher rate of OA in the JAKi group might have influenced the poorer drug survival. Additionally, it should also be noted that the amount of available data (e.g. DAPSA) was different in those groups. Furthermore, the extent of cutaneous involvement was not considered in our analysis but can have a substantial impact on quality of life and thereby can influence drug selection and potentially drug survival in PsA (22). We could not analyze dermatologic data such as the Psoriasis Area and Severity Index (PASI) because only rheumatologic practices provided data in the RHADAR database, and the PASI is not routinely assessed in rheumatologic routine care. It is conceivable that IL-17i showed these considerable survival rates due to the ability of these drugs to address both skin and joint involvement adequately. Head-to-head studies against TNFi suggest that IL-17i may have higher levels of effectiveness in combined skin and joint diseases (23–25).

Our study population was highly comparable to the general German PsA population reported from healthcare insurance data based on similar mean age (53.8 years in our study vs. 58.9 years) and similar proportions of females (55% vs. 63%) (26). Although DAPSA as a parameter of disease activity is not available for the general German PsA population, a smaller study (n=104) in German outpatients (with similar age and sex distribution) reported lower DAPSA mean (SD) scores of 10.5 (10.6) than observed here (17.9 [16.3]) (27). Given the high standard deviation for this parameter in both studies, this reported difference is likely not significant. Thus, we conclude that our study population generally represents the German PsA population, and our findings may be transferable to all German PsA patients.

The drug survival rates reported here are consistent with drug survival times reported in recently published analyses of other PsA registries (5, 8, 19, 28). However, these other analyses evaluated retention for individual drugs but did not group their analyses into drug classes based on MoA . The only German real-world analysis on drug survival among a small cohort of 348 PsA patients presented overall 1-year persistence rates of 57.5% for all bDMARDs, which is lower than those observed in this study (29). To date, there is only minimal information available on real-world drug retention of JAKi in PsA (5, 19).

The aforementioned registry studies also reported Cox regression models to analyze differences in drug retention. In the Danish DANBIO registry, ustekinumab, tofacitinib, and infliximab had the lowest adjusted HR for persistence, while TNFi and IL-17i were superior and had similar HR (5). These results align with our reported data, indicating that IL-12/23i and JAKi have lower survival rates and a lower HR for persistence than IL-17i and TNFi. A large compound North European registry study also showed no difference in HRs between IL-17i and TNFi (8). Interestingly, the only real-world German study showed that ustekinumab had the highest HR for 1-year drug persistence (29). However, these data might not be directly comparable due to the smaller case number (n=348), with only 32 patients receiving ustekinumab, and the different analysis period, which was shorter and earlier (2014–2017) compared with our study (29). Based on these other studies, we consider it likely that our reported data realistically depict the actual outpatient PsA treatment situation with bDMARDs and tsDMARDs in the German healthcare sector in the JAKi era.

### Strengths and limitations

The findings of our study have several limiting factors. First, it is a retrospective analysis of a real-world clinical registry; therefore, drug survival was not evaluated prospectively. The RHADAR database is a registry with automatic data transfer from rheumatologic practices; these data are not monitored, which might explain the partly high rate of missing values. Furthermore, not all available disease parameters were assessed in all patients, dermatologic parameters were not reported, and reasons for discontinuation were not provided. Although the database included clinician-prescribed therapies, adherence to treatments was not assessed. Because of the retrospective, registry-based study design, groups were not matched or stratified. All patients from the registry with a diagnosis of PsA and a new prescription for a bDMARD or JAKi were included. Our analyses clustered substances due to their MoA and we did not distinguish between different agents within a drug class; other studies have reported small differences in drug survival among agents in a specific drug class (5). This effect might be more relevant for TNFi than other drug classes due to the greater number of agents in the TNFi drug class. We did not distinguish between axial and peripheral phenotypes of PsA. Others have reported that patients with axial involvement tend to have a poorer response to IL-12/23i than to TNFi or IL-17i (30).

The major strength of our study lies in its multicentric nature and its focus on real-world performance, which highlights the challenges of conducting drug survival studies among outpatients due to high costs and the complexities of integrating study activities into routine outpatient care. Despite these difficulties, such studies are crucial as they offer a cross-sectional analysis of actual care in rheumatology practices and can potentially influence economic decision-making in the healthcare sector. Additionally, our study contributes novel real-world data on drug retention, specifically JAKi in PsA, a currently underrepresented topic in existing research. Our data support the hypothesis that bDMARDs and JAKi are associated with different drug survival rates in PsA.

## Conclusion

Even in the JAKi era, TNFi agents are still the most commonly prescribed new bDMARD/tsDMARD treatment in PsA in the German outpatient sector compared with IL-17i, IL-12/23i, and JAKi. IL-17i and TNFi are more likely to persist longer than JAKi and IL-12/23i in these patients. At least for JAKi-treated patients, lower drug survival rates could be due to more severe disease with a higher number of pretreatments and a higher rate of OA compared with TNFi or to evolving safety data and treatment recommendations for this drug class. The higher survival rates associated with IL-17i might be influenced by a lower prevalence of OA in this group compared to JAKi but were not related to more favorable patient or disease characteristics and may therefore reflect simultaneous effective control of skin and joint involvement.

## Data availability statement

The original contributions presented in the study are included in the article/**Supplementary Material**. Further inquiries can be directed to the corresponding author.

## Ethics statement

The study involving humans were submitted by Medizinische Ethikkommission an der Julius-Maximilians-Universität Würzburg, Josef-Schneider-Str. 4 97080, Würzburg. The study were conducted in accordance with the local legislation and institutional requirements. The participants provided their written informed consent to participate in this study.

## Author contributions

P-PS: Conceptualization, Data curation, Formal analysis, Investigation, Methodology, Visualization, Writing – original draft, Writing – review & editing. ME: Conceptualization, Data curation, Formal analysis, Investigation, Methodology, Software, Visualization, Writing – original draft, Writing – review & editing. LR: Conceptualization, Investigation, Methodology, Writing – original draft, Writing – review & editing. TW: Conceptualization, Investigation, Methodology, Writing – original draft, Writing – review & editing. MF: Writing – original draft, Writing – review & editing. MS: Writing – original draft, Writing – review & editing. MG: Writing – original draft, Writing – review & editing. AS: Writing – original draft, Writing – review & editing. PB-B: Writing – original draft, Writing – review & editing. CD: Writing – original draft, Writing – review & editing. KK: Writing – original draft, Writing – review & editing. GG: Writing – original draft, Writing – review & editing. PW: Writing – original draft, Writing – review & editing. SS-M: Writing – original draft, Writing – review & editing. CK: Writing – original draft, Writing – review & editing. WV: Writing – original draft, Writing – review & editing. JH: Writing – original draft, Writing – review & editing. MW: Writing – review & editing, Writing – original draft. SK: Conceptualization, Data curation, Formal analysis, Funding acquisition, Investigation, Methodology, Project administration, Software, Supervision, Writing – original draft, Writing – review & editing.
