# Supplementary material for: Drug survival superiority of tumor necrosis factor inhibitors and interleukin-17 inhibitors over Janus kinase inhibitors and interleukin-12/23 inhibitors in German psoriatic arthritis outpatients: retrospective analysis of the RHADAR database
Source: Front Immunol. 2024 May 23;15:1395968. doi: 10.3389/fimmu.2024.1395968 (PMC11153701; doi:10.3389/fimmu.2024.1395968)
Supplement: Supplementary file 1 [file Table_1.docx]

**Supplementary File to** “**Drug survival superiority of tumor necrosis factor inhibitors and interleukin-17 inhibitors over Janus kinase inhibitors and interleukin-12/23 inhibitors in German psoriatic arthritis outpatients: Retrospective analysis of the RHADAR database.”**

**Authors:** Patrick-Pascal Strunz MD^1 *^, Matthias Englbrecht PhD^2^, Linus Maximilian Risser MD^3^, Torsten Witte MD^3^, Matthias Froehlich MD^1^, Marc Schmalzing MD^1^, Michael Gernert MD^1^, Astrid Schmieder MD^4^, Peter Bartz-Bazzanella MD^5, 6^, Cay von der Decken MD^5, 6, 11^, Kirsten Karberg MD^7^, Georg Gauler MD^8^, Patrick Wurth MD^8^, Susanna Späthling-Mestekemper MD^9^, Christoph Kuhn MD ^10^, Wolfgang Vorbrüggen MD^11^, Johannes Heck^12^, Martin Welcker MD^11, 13^ and Stefan Kleinert MD^1, 14^

^1^ University Hospital of Wuerzburg, Departement of Medicine II, Rheumatology/ Clinical Immunology, Würzburg, Germany

^2^ Freelance Healthcare Data Scientist, Greven, Germany

^3^Medical School Hannover, Department of Rheumatology and Immunology, Hannover, Germany

^4^Clinic for Dermatology, Venereology and Allergology, University Hospital Wuerzburg, Germany

^5^Klinik für Internistische Rheumatologie, Rhein-Maas-Klinikum, Würselen, Germany

^6^Medizinisches Versorgungszentrum, Stolberg, Germany

^7^Rheumatologisches Versorgungszentrum Steglitz, Berlin, Germany

^8^ Rheumatology Practice, Osnabrück, Germany

^9^ Rheumapraxis München¸ Germany

^10^ Praxis für Rheumatologie, Karlsruhe, Germany

^11^Verein zur Förderung der Rheumatologie e.V., Würselen, Germany

^12^ Hannover Medical School, Institute for Clinical Pharmacology, Hannover, Germany

^13^ Medizinisches Versorgungszentrum für Rheumatologie Dr. M. Welcker GmbH, Planegg, Germany

^14^ Praxisgemeinschaft Rheumatologie-Nephrologie, Erlangen, Germany

***Correspondence:**

Dr. med. Patrick-Pascal Strunz

University Hospital of Wuerzburg,

Departement of Medicine II, Rheumatology/ Clinical Immunology,

Oberdürrbacher Straße 6

97080 Würzburg, Germany

Strunz_p@ukw.de

**S1. Characteristics of the subpopulations stratified by treatment MoA**

| Patient characteristics of the IL12/23i-subpopulation | | | | | | | | | | |
| --- | --- | --- | --- | --- | --- | --- | --- | --- | --- | --- |
|  | **Valid (n)** | **Valid (%)** | **Mean** | **95%CI (lower)** | **95%CI (upper)** | **SD** | **SEM** | **Median** | **25% Quantile** | **75% Quantile** |
| Gender (male) | 9 | 18.0 | NA | NA | NA | NA | NA | NA | NA | NA |
| Gender (female) | 41 | 82.0 | NA | NA | NA | NA | NA | NA | NA | NA |
| Age | 50 | 100.0 | 54.1 | 51.3 | 56.8 | 9.9 | 1.4 | 55.0 | 49.0 | 60.8 |
| Disease duration (years) | 43 | 86.0 | 13.3 | 9.8 | 16.9 | 11.8 | 1.8 | 8.0 | 5.5 | 16.5 |
| DAPSA | 16 | 32.0 | 21.9 | 15.4 | 28.3 | 13.1 | 3.3 | 20.3 | 15.1 | 25.6 |
| TJC (DAPSA) | 28 | 56.0 | 9.3 | 6.1 | 12.5 | 8.7 | 1.6 | 7.5 | 2.8 | 12.0 |
| SJC (DAPSA) | 28 | 56.0 | 2.8 | 1.6 | 4.0 | 3.3 | 0.6 | 2.0 | 0.0 | 5.0 |
| ESR (mm/h) | 20 | 40.0 | 19.5 | 11.7 | 27.3 | 17.9 | 4.0 | 16.5 | 7.8 | 22.0 |
| CRP (mg/dl) | 23 | 46.0 | 0.7 | 0.4 | 1.0 | 0.7 | 0.2 | 0.3 | 0.3 | 0.9 |
| Disease activity - patient (0-100) | 22 | 44.0 | 46.5 | 36.1 | 56.8 | 24.8 | 5.3 | 53.0 | 30.0 | 60.0 |
| Pain (0-100) | 20 | 40.0 | 45.0 | 36.2 | 53.7 | 19.9 | 4.4 | 51.0 | 30.0 | 60.5 |
| Morning stiffness (min) | 19 | 38.0 | 55.8 | 28.8 | 82.9 | 60.2 | 13.8 | 30.0 | 17.0 | 73.5 |
| FFbH | 30 | 60.0 | 70.5 | 63.5 | 77.5 | 19.7 | 3.6 | 72.2 | 60.4 | 83.3 |

| Patient characteristics of the IL17i-subpopulation | | | | | | | | | | |
| --- | --- | --- | --- | --- | --- | --- | --- | --- | --- | --- |
|  | **Valid (n)** | **Valid (%)** | **Mean** | **95%CI (lower)** | **95%CI (upper)** | **SD** | **SEM** | **Median** | **25% Quantile** | **75% Quantile** |
| Gender (male) | 137 | 38.1 | NA | NA | NA | NA | NA | NA | NA | NA |
| Gender (female) | 223 | 61.9 | NA | NA | NA | NA | NA | NA | NA | NA |
| Age | 360 | 100.0 | 54.5 | 53.3 | 55.7 | 11.7 | 0.6 | 55.0 | 47.0 | 62.0 |
| Disease duration (years) | 320 | 88.9 | 10.5 | 9.5 | 11.5 | 9.2 | 0.5 | 8.0 | 4.0 | 15.0 |
| DAPSA | 108 | 30.0 | 17.9 | 15.5 | 20.3 | 12.6 | 1.2 | 15.4 | 9.9 | 23.5 |
| TJC (DAPSA) | 223 | 61.9 | 6.2 | 5.1 | 7.3 | 8.5 | 0.6 | 3.0 | 1.0 | 8.0 |
| SJC (DAPSA) | 223 | 61.9 | 2.7 | 2.1 | 3.3 | 4.3 | 0.3 | 1.0 | 0.0 | 4.0 |
| ESR (mm/h) | 185 | 51.4 | 15.9 | 13.8 | 18.0 | 14.6 | 1.1 | 12.0 | 6.0 | 20.0 |
| CRP (mg/dl) | 198 | 55.0 | 0.5 | 0.4 | 0.7 | 1.0 | 0.1 | 0.3 | 0.1 | 0.4 |
| Disease activity - patient (0-100) | 215 | 59.7 | 44.1 | 41.1 | 47.1 | 22.4 | 1.5 | 48.0 | 30.0 | 60.0 |
| Pain (0-100) | 181 | 50.3 | 43.4 | 39.7 | 47.0 | 25.1 | 1.9 | 46.0 | 23.0 | 60.0 |
| Morning stiffness (min) | 172 | 47.8 | 90.9 | 58.9 | 123.0 | 214.5 | 16.4 | 30.0 | 20.8 | 62.5 |
| FFbH | 227 | 63.1 | 73.5 | 70.7 | 76.3 | 21.7 | 1.4 | 77.8 | 55.6 | 91.7 |

| Patient characteristics of the IL23i-subpopulation | | | | | | | | | | |
| --- | --- | --- | --- | --- | --- | --- | --- | --- | --- | --- |
|  | **Valid (n)** | **Valid (%)** | **Mean** | **95%CI (lower)** | **95%CI (upper)** | **SD** | **SEM** | **Median** | **25% Quantile** | **75% Quantile** |
| Gender (male) | 13 | 41.9 | NA | NA | NA | NA | NA | NA | NA | NA |
| Gender (female) | 18 | 58.1 | NA | NA | NA | NA | NA | NA | NA | NA |
| Age | 31 | 100.0 | 54.7 | 50.7 | 58.7 | 11.5 | 2.1 | 56.0 | 48.0 | 63.0 |
| Disease duration (years) | 31 | 100.0 | 10.8 | 8.0 | 13.5 | 7.9 | 1.4 | 8.0 | 6.0 | 14.5 |
| DAPSA | 12 | 38.7 | 15.5 | 6.2 | 24.9 | 16.5 | 4.8 | 10.6 | 4.5 | 23.0 |
| TJC (DAPSA) | 19 | 61.3 | 5.0 | 1.2 | 8.8 | 8.4 | 1.9 | 1.0 | 0.0 | 4.5 |
| SJC (DAPSA) | 19 | 61.3 | 1.4 | 0.0 | 2.8 | 3.1 | 0.7 | 0.0 | 0.0 | 1.0 |
| ESR (mm/h) | 13 | 41.9 | 8.2 | 5.0 | 11.4 | 5.9 | 1.6 | 6.0 | 4.0 | 8.0 |
| CRP (mg/dl) | 18 | 58.1 | 0.3 | 0.2 | 0.5 | 0.3 | 0.1 | 0.2 | 0.1 | 0.4 |
| Disease activity - patient (0-100) | 19 | 61.3 | 41.2 | 29.5 | 52.9 | 26.1 | 6.0 | 30.0 | 20.0 | 60.0 |
| Pain (0-100) | 13 | 41.9 | 30.1 | 14.4 | 45.8 | 28.9 | 8.0 | 20.0 | 10.0 | 50.0 |
| Morning stiffness (min) | 13 | 41.9 | 158.2 | -54.0 | 370.3 | 390.3 | 108.3 | 30.0 | 30.0 | 45.0 |
| FFbH | 19 | 61.3 | 79.1 | 68.3 | 89.9 | 24.1 | 5.5 | 91.7 | 69.4 | 97.2 |

| Patient characteristics of the JAKi-subpopulation | | | | | | | | | | |
| --- | --- | --- | --- | --- | --- | --- | --- | --- | --- | --- |
|  | **Valid (n)** | **Valid (%)** | **Mean** | **95%CI (lower)** | **95%CI (upper)** | **SD** | **SEM** | **Median** | **25% Quantile** | **75% Quantile** |
| Gender (male) | 41 | 29.9 | NA | NA | NA | NA | NA | NA | NA | NA |
| Gender (female) | 96 | 70.1 | NA | NA | NA | NA | NA | NA | NA | NA |
| Age | 137 | 100.0 | 57.1 | 55.3 | 58.9 | 10.8 | 0.9 | 56.0 | 51.0 | 64.0 |
| Disease duration (years) | 114 | 83.2 | 11.6 | 9.7 | 13.5 | 10.4 | 1.0 | 8.0 | 4.0 | 15.0 |
| DAPSA | 77 | 56.2 | 21.8 | 19.0 | 24.7 | 12.8 | 1.5 | 19.3 | 14.3 | 26.4 |
| TJC (DAPSA) | 109 | 79.6 | 7.6 | 6.3 | 8.8 | 6.7 | 0.6 | 6.0 | 4.0 | 10.0 |
| SJC (DAPSA) | 109 | 79.6 | 3.3 | 2.5 | 4.0 | 4.1 | 0.4 | 2.0 | 0.0 | 5.0 |
| ESR (mm/h) | 79 | 57.7 | 16.1 | 12.9 | 19.4 | 14.6 | 1.6 | 12.0 | 6.0 | 20.0 |
| CRP (mg/dl) | 105 | 76.6 | 0.6 | 0.4 | 0.8 | 1.1 | 0.1 | 0.3 | 0.3 | 0.5 |
| Disease activity - patient (0-100) | 96 | 70.1 | 48.3 | 44.3 | 52.3 | 20.1 | 2.1 | 50.0 | 39.5 | 60.0 |
| Pain (0-100) | 72 | 52.6 | 46.1 | 41.0 | 51.2 | 22.1 | 2.6 | 46.0 | 33.0 | 59.2 |
| Morning stiffness (min) | 65 | 47.4 | 59.5 | 46.2 | 72.9 | 54.8 | 6.8 | 52.0 | 25.0 | 63.0 |
| FFbH | 89 | 65.0 | 72.4 | 68.2 | 76.6 | 20.3 | 2.2 | 75.0 | 58.3 | 88.9 |

| Patient characteristics of the TNFi-subpopulation | | | | | | | | | | |
| --- | --- | --- | --- | --- | --- | --- | --- | --- | --- | --- |
|  | **Valid (n)** | **Valid (%)** | **Mean** | **95%CI (lower)** | **95%CI (upper)** | **SD** | **SEM** | **Median** | **25% Quantile** | **75% Quantile** |
| Gender (male) | 296 | 38.2 | NA | NA | NA | NA | NA | NA | NA | NA |
| Gender (female) | 477 | 61.6 | NA | NA | NA | NA | NA | NA | NA | NA |
| Age | 774 | 100.0 | 52.8 | 51.9 | 53.7 | 12.9 | 0.5 | 54.0 | 45.0 | 62.0 |
| Disease duration (years) | 681 | 88.0 | 10.1 | 9.4 | 10.8 | 9.2 | 0.4 | 7.0 | 3.0 | 14.0 |
| DAPSA | 240 | 31.0 | 16.5 | 14.1 | 18.9 | 18.7 | 1.2 | 14.4 | 5.3 | 22.5 |
| TJC (DAPSA) | 476 | 61.5 | 5.0 | 4.4 | 5.6 | 6.5 | 0.3 | 3.0 | 0.0 | 8.0 |
| SJC (DAPSA) | 476 | 61.5 | 2.2 | 1.9 | 2.5 | 3.3 | 0.1 | 1.0 | 0.0 | 3.0 |
| ESR (mm/h) | 405 | 52.3 | 15.5 | 14.1 | 17.0 | 14.5 | 0.7 | 11.0 | 6.0 | 20.0 |
| CRP (mg/dl) | 435 | 56.2 | 0.9 | -0.1 | 2.0 | 10.9 | 0.5 | 0.3 | 0.1 | 0.3 |
| Disease activity - patient (0-100) | 440 | 56.8 | 39.2 | 36.8 | 41.5 | 25.2 | 1.2 | 40.0 | 20.0 | 60.0 |
| Pain (0-100) | 355 | 45.9 | 37.3 | 34.5 | 40.1 | 26.8 | 1.4 | 37.0 | 11.0 | 60.0 |
| Morning stiffness (min) | 343 | 44.3 | 57.1 | 43.4 | 70.9 | 130.0 | 7.0 | 30.0 | 12.0 | 60.0 |
| FFbH | 496 | 64.1 | 78.4 | 76.5 | 80.3 | 21.1 | 0.9 | 83.3 | 66.7 | 97.2 |

**S2. Concomitant treatment stratified by treatment MoA**

1. **IL-17i vs. IL-12/23i**

|  | **Monotherapy** | **Combination therapy** | **Total** |
| --- | --- | --- | --- |
| **IL-17i** | 280 | 80 | 360 |
| **IL-12/23i** | 37 | 13 | 50 |
| **Total** | 317 | 93 | 410 |

Χ², df: 0.3573, 1; OR: 1.230; 95% CI: 0.6235 to 2.425; p= 0.5500

1. **IL-17i vs. JAKi**

|  | **Monotherapy** | **Combination therapy** | **Total** |
| --- | --- | --- | --- |
| **IL-17i** | 280 | 80 | 360 |
| **JAKi** | 109 | 28 | 137 |
| **Total** | 389 | 108 | 497 |

Χ², df: 0.1857, 1; OR: 0.8991; 95% CI: 0.5541 to 1.459; p= 0.6665

1. **TNFi vs. IL-12/23i**

|  | **Monotherapy** | **Combination therapy** | **Total** |
| --- | --- | --- | --- |
| **TNFi** | 536 | 238 | 774 |
| **IL-12/23i** | 37 | 13 | 50 |
| **Total** | 573 | 251 | 824 |

Χ², df: 0.5001, 1; OR: 0.7913; 95% CI: 0.4130 to 1.516; p= 0.4794

1. **TNFi vs. JAKi**

|  | **Monotherapy** | **Combination therapy** | **Total** |
| --- | --- | --- | --- |
| **TNFi** | 536 | 238 | 774 |
| **JAKi** | 109 | 28 | 137 |
| **Total** | 645 | 266 | 911 |

Χ², df: 5.987, 1; OR: 0.5785; 95% CI: 0.3716 to 0.9006; p= 0.0144

**S3. Osteoarthritis stratified by treatment MoA**

1. **IL-17i vs. IL-12/23i**

|  | **Osteoarthritis** | **No osteoarthritis** | **Total** |
| --- | --- | --- | --- |
| **IL-17i** | 88 | 272 | 360 |
| **IL-12/23i** | 29 | 21 | 50 |
| **Total** | 117 | 293 | 410 |

Χ², df: 24.24, 1; OR 0.2343; 95% CI: 0.1272 to 0.4316; p<0.001

1. **IL-17i vs. JAKi**

|  | **Osteoarthritis** | **No osteoarthritis** | **Total** |
| --- | --- | --- | --- |
| **IL-17i** | 88 | 272 | 360 |
| **JAKi** | 50 | 87 | 137 |
| **Total** | 138 | 359 | 497 |

Χ², df: 7.186, 1; OR: 0.5629; 95% CI: 0.3688 to 0.8593; p= 0.0073

1. **TNFi vs. IL-12/23i**

|  | **Osteoarthritis** | **No Osteoarthritis** | **Total** |
| --- | --- | --- | --- |
| **TNFi** | 178 | 596 | 774 |
| **IL-12/23i** | 29 | 21 | 50 |
| **Total** | 207 | 617 | 824 |

Χ², df: 30.59, 1; OR: 0.2163; 95% CI: 0.1203 to 0.3887; p<0.001

1. **TNFi vs. JAKi**

|  | **Osteoarthritis** | **No Osteoarthritis** | **Total** |
| --- | --- | --- | --- |
| **TNFi** | 178 | 596 | 774 |
| **JAKi** | 50 | 87 | 137 |
| **Total** | 228 | 683 | 911 |

Χ², df: 11.30, 1; OR: 0.5197; 95% CI: 0.3532 to 0.7646; p= 0.0008
